# Peripheral anterior chamber depth and screening techniques for primary angle closure disease in community elderly Chinese

**DOI:** 10.1186/s12886-020-01618-3

**Published:** 2020-08-28

**Authors:** Qin Luo, Wenwen Xue, Yin Yuan, Chaowei Fu, Jiangnan He, Haidong Zou, Xiaowei Tong, Richard K. Lee, Ling Ge

**Affiliations:** 1 Department of Ophthalmology, Shanghai Eye Disease Prevention & Treatment Center/ Shanghai Eye Hospital, No. 380 Kangding Road, Shanghai, 200040 China; 2 Department of Ophthalmology, Shanghai General Hospital, Shanghai Jiao Tong University School of Medicine, No. 100, Haining Road, Shanghai, 200040 China; 3 Department of Social Medicine, School of Public Health, Key Laboratory of Public Health Safety, NHC Key Laboratory of Health Technology Assessment, Fudan University, Shanghai, 200032 China; 4 Bascom Palmer Eye Institute, 900 N.W. 17th Street, Miami, FL 33136 USA

**Keywords:** Peripheral anterior chamber depth, Primary angle closure disease, Screening, Chinese

## Abstract

**Background:**

To investigate the distribution of peripheral anterior chamber depth (ACD) and the relationship between peripheral ACD and gonioscopy compared to other ocular parameters for primary angle closure disease (PACD) screening. We performed a population-based survey in Pudong New District of Shanghai, China, in 2011.

**Methods:**

Cross-sectional study. Adults 50 and older were enrolled from a population-based study using cluster random sampling in Pudong New District, Shanghai. Remote ocular screening was performed with digital anterior eye structure photography. Van Herrick measurements were used to evaluate the peripheral ACD, the depth of the peripheral anterior chamber, and corneal thickness (CT), and the ACD to CT ratio was calculated. Subjects with peripheral ACD less than 0.5 CT were made follow-up appointments for clinical examination with gonioscopy. Receiver operating characteristic curves (ROC) were generated to show the performance of different tests in screening for primary angle closure disease (PACD).

**Results:**

Two thousand five hundred twenty-eight adults participated in the study with 91 patients diagnosed with PACD. Two thousand four hundred sixty-three subjects had valid data in the right eye available for analysis. The mean peripheral ACD values for men and women were significantly different: 1.04 ± 0.46 (range 0.11–2.93) CT and 0.87 ± 0.41 (range 0.12–2.96) CT respectively (t = − 4.18; *P*<0.0001). Multivariate logistic regression analysis reveals that peripheral ACD declined by 0.31 CT (*P* < 0.0001) per diopter of SE and was 0.19 CT (*P* < 0.0001) shallower in women than in men (r2 = 0.1304, *P* < 0.0001). Peripheral ACD performed best in screening for PACD.

**Conclusions:**

Peripheral ACD measurement is recommended for PACD screening in community elderly Chinese.

## Background

Glaucoma is the most common cause of irreversible blindness worldwide, affecting about 67 million people [1–3]. Primary angle closure glaucoma (PACG) is the major type of glaucoma in Asia and is a significant cause of global visual morbidity [4–8]. PACG is higher in Asians than Europeans and Africans, with over 80% of PACG worldwide cases in Asia. PACG affects approximately 0.75% adult Asians, doubling in incidence per decade, and 60% of patients are female. The prevalence rates vary greatly by ethnic region and has been calculated to be approximately 1.10% (0.85, 1.44) in the Chinese population [9]. The rate of blindness caused by PACG is high in mainland China. Random effect model meta-analysis results show the overall blindness rate was 38.3% [95%CI (28.1, 49.6%)] [10]. A means of detecting those at risk (people with occludable anterior chamber angles) is a prerequisite for a primary angle closure glaucoma prevention program.

Though gonioscopy is recognized as the gold standard for identifying individuals at risk for primary angle closure disease (PACD), this technique requires highly trained personnel and expensive equipment such as a slit lamp (along with gonioscopy lenses), which are in short supply in remote districts in China. Moreover, gonioscopy is relatively subjective, making it less than ideal for comparisons of prevalence between regions [11]. Gonioscopy requires ocular contact examination which may affect the informed consent rate for community screening.

Eyes with PACD tend to have certain biometric characteristics. These include shallow anterior chamber depth (ACD), thick lens, anterior lens position, small corneal diameter and radius of curvature, and short axial length [12–14]. Among these parameters, shallow ACD is known as a key risk factor in most ethnic groups for PACD [15–17].

Some studies suggest that this may not be true for central ACD in East Asian people, where the role of non-pupillary block angle closure in relatively deep anterior chambers has been debated [15, 18]. Peripheral ACD has proved to be significantly associated with primary angle closure (PAC) and may reflect a non-pupillary block risk factor for PAC to some extent [16].

We performed a population-based survey in Pudong New District of Shanghai, China, in 2011 [19]. 2528 cases were examined and 91 PACD were diagnosed (prevalence rate 3.6%), including 6 PACG (prevalence rate 0.24%), 9 PAC (prevalence rate 0.36%) and 76 primary angle closure suspects (PACS, prevalence rate 3.01%) [20]. The goal of this study was to report the distribution of peripheral ACD and its association with age, gender, refraction, and intraocular pressure (IOP) in elderly Chinese people. The relationship between peripheral ACD and gonioscopy was investigated, assessing different methods (peripheral ACD, spherical equivalent [SE], intraocular pressure [IOP]) for PACD screening in community. Peripheral ACD performed best in screening for PACD.

## Methods

### Investigation place and targets

This study assessed the Huamu community in Pudong New District of Shanghai. This community has a history of more than 30 years and is home to a stable population. The social and economic levels in the Huamu community are at the average level of Shanghai.

This study was performed in accordance with the Declaration of Helsinki and was approved by the Ethics Committee of Shanghai General Hospital Affiliated to Shanghai Jiaotong University (registration number: 2010 K059). All subjects signed informed consents before being examined.

### Sampling

The detailed study protocol has been described elsewhere [19]. According to the preceding criteria, a total of 3146 people aged 50 and older were enrolled from a population-based study using cluster random sampling in Pudong New District, Shanghai. In the current study, only data from right eyes were analyzed and exclusion criteria include previous cataract surgery and other intraocular operations, ocular trauma, intraocular inflammation, iris dysplasia, atrophic eyeball, and incomplete data.

### Investigation procedure

This research adopted remote screening in the community in combination with subsequent clinical evaluation and diagnosis at a tertiary eye hospital. Subject identities (IDs) were verified and personal information was collected. Remote screening was then performed, including visual acuity, refraction, IOP measurement, slit lamp digital anterior eye structure photography, and digital fundus photography. The collected information was then transmitted to the Shanghai Eye Disease Prevention and Treatment Center through a dedicated network. The ophthalmologists clinically experienced in glaucoma diagnosis reviewed the photographs and gathered data. The investigation team then made an appointment for examination at the Shanghai Eye Disease Prevention and Treatment Center for glaucoma suspects after the preliminary check. Re-examinations included IOP measurement, gonioscopy, perimetry testing by Humphrey automated perimetry, and retinal nerve fiber layer (RNFL) thickness measurement by optical coherence tomography (OCT).

### Ophthalmic assessment

Visual acuity was measured using a standard illuminated LogMAR (minimum angle of resolution) E chart (Precision Vision, IL, USA), and the presenting visual acuity and the best corrected visual acuity (BCVA) was recorded. The autorefraction data were converted to the spherical equivalent (SE: sphere + 1/2 cylinder).

Digital anterior segment slit lamp photographs were taken in a dark room. Abnormalities of the anterior segment, such as corneal opacity, iris atrophy, pupil size, lens status, presence of glaucomflecken and turbidity of the crystalline lens were recorded. The peripheral ACD around the limbus on the temporal side using an illuminated slit lamp, which casts a clear line on the iris. As described in our previous study [19], peripheral ACD was described as a percentage of corneal thickness at the temporal limbus with the slit beam directed perpendicular to the ocular surface (The brightest, narrowest illumination beam was used. The illumination column was offset from the microscope axis by 40 °).

Centered by the optic disc and macula, two digital fundus photographs were taken using a digital nonmydriatic fundus camera (CanonCR-DGi, Japan).

All the data collected remotely were transmitted to the Shanghai Eye Disease Prevention and Treatment Center, and the film reading doctors used the Van Herrick method to evaluate the peripheral ACD. Microsoft Paint was used to measure the depth of the peripheral anterior chamber and the corresponding corneal thickness (CT) in the anterior segment photograph, and the ACD to the CT ratio was calculated. Three measurements were carried out and recorded, and the median of 3 readings was used to analyze. A modified Van Herrick grading scheme was used in this study with eight categories (0, 0.05, 0.15, 0.25, 0.35, 0.45, 0.75, and ≥ 1.0 CT) instead of the usual five categories (0, 1/4, 1/2, 3/4, 1 CT). These values were chosen to give class limits of 0, < 0.1, < 0.2, < 0.3, < 0.4, < 0.5, < 1.0, and ≥ 1.0 CT. All subjects with peripheral ACD less than 0.5 CT were made an appointment for gonioscopy in the tertiary care eye hospital. Microsoft Paint was also used to measure the vertical diameters of the optic cup and optic disk, and vertical cup to disk ratio (VCDR) was calculated. Disk hemorrhage, optic nerve head notching, and other abnormal characteristics in the fundus photograph were recorded.

Gonioscopy was performed with a Goldman one-mirror lens (HaggStreit, Bern, Switzerland) at 16× magnification using a 1-mm-long slit with low ambient illumination to prevent light from irradiating the pupil area. A vertically oriented light beam was used for observing both the superior and the inferior anterior chamber angles and horizontally for the nasal and temporal quadrants. The anterior chamber angle was first evaluated statically, and then dynamically with the lens was performed. The anterior chamber angles were characterized with the Spaeth grading system. One senior doctor did gonioscopy for all participants in the hospital.

SITA-FAST 30–2 mode white-on-white automated perimetry (Humphrey 720, Carl Zeiss Meditec, CA, USA) was performed with refractive correction. RNFL thickness was measured using Cirrus spectral domain HD-OCT (Carl Zeiss Meditec, CA, USA).

### Diagnostic definitions

Glaucoma suspects were identified according to presence of any of the following signs: VCDR > 0.5 in either eye, VCDR asymmetry ≥0.2, or a neuroretinal rim width reduced to < 0.1 CDR (between 11 and 1 o’clock or 5 and 7 o’clock), optic disk hemorrhage, notching in the optic disc rim or RNFL defects on the superior or inferior temporal near the disc in the fundus photograph, or IOP ≥21 mmHg [19].

Glaucoma cases were diagnosed using ISGEO criteria [21]. Glaucoma was identified in accordance with three levels of evidence. The division of glaucoma into PACG versus primary open angle glaucoma (POAG) was based on gonioscopic finding of a narrow angle. PACS was defined as an eye with appositional contact between the peripheral iris and posterior trabecular meshwork [21]. In epidemiological research, a narrow angle has most often been defined as an angle in which > 270° of the posterior trabecular meshwork (the part which is often pigmented) cannot be seen during a static examination. PAC was regarded as an eye with an occludable drainage angle and features indicating that trabecular obstruction by the peripheral iris have occurred, such as peripheral anterior synechiae (PAS), elevated IOP, iris whorling (distortion of the radially orientated iris fibers), “glaucomfleken” lens opacities, or excessive pigment deposition on the trabecular surface and and the optic disc does not have glaucomatous damage [21].

### Statistical analysis

A database was established with EpiData 3.0 (EpiData Association, Odense, Denmark). Statistical analysis was performed using SAS version 9.1.3 (SAS Inc., NC, USA). The 95% confidence interval (CI) was calculated assuming a normal distribution. This study first performed univariate logistic regression analysis on the factors influencing peripheral ACD, and then performed multivariate logistic regression analysis to explore the association of age, gender, IOP, and refraction with peripheral ACD. A value of *P* < 0.05 was defined as statistically significant.

## Results

**Fig. 1:**
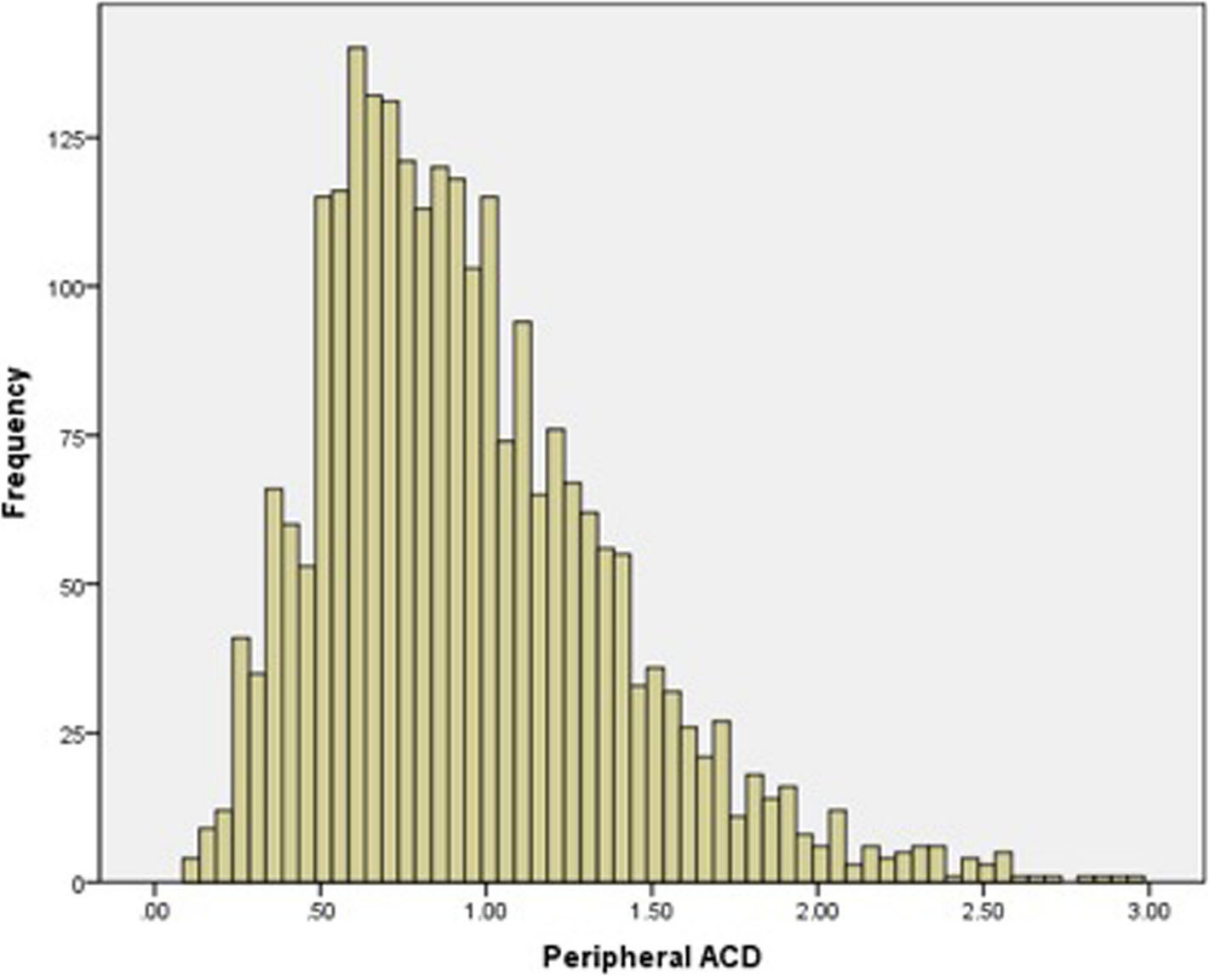
Histogram showing distribution of peripheral ACD (mean, 0.94 CT; standard deviation, 0.44 CT) in 2463 right eyes

A total of 2528 subjects of the screened 3146 adults participated in the study with valid data, giving a response rate of 80.36% [20]. Sixty-five cases were excluded in the study because of previous intraocular surgery, ocular trauma, intraocular inflammation, iris dysplasia, atrophic eyeball, and/or incomplete data in right eye. Two thousand four hundred sixty-three subjects had valid data in right eye available for analysis (Fig. 1).

The mean peripheral ACD values for men and women were significantly different: 1.04 ± 0.46 (range 0.11–2.93) CT and 0.87 ± 0.41 (range 0.12–2.96) CT respectively (*t* = − 4.18; *P*<0.0001; Table 1). Older subjects had shallower peripheral ACD, observed in both men and women and all subjects (*F* = 4.54; *P* = 0.0035; Table 1), except in the oldest group (80–94 years old). The peripheral ACD between age group 50–60 and age group 70–80 were significantly different.

**Table 1:** Characteristic of peripheral ACD in right eyes

	n	Mean ± SD	Median	25th Percentile	50th Percentile	75th Percentile	Missing	Range
**Men (age)**								
50–60	368	1.06 ± 0.47	1.00	0.72	1.00	1.37	0	0.18–2.93
60–70	398	1.02 ± 0.46	0.94	0.69	0.94	1.30	0	0.11–2.81
70–80	218	1.00 ± 0.44	0.94	0.67	0.94	1.24	0	0.21–2.60
80–94	52	1.11 ± 0.59	1.02	0.65	1.02	1.29	0	0.28–2.72
All	1036	1.04 ± 0.46	0.97	0.69	0.97	1.30	0	0.11–2.93
**Women (age)**								
50–60	610	0.91 ± 0.40	0.85	0.63	0.85	1.11	0	0.12–2.65
60–70	497	0.84 ± 0.40	0.79	0.54	0.79	1.08	0	0.13–2.33
70–80	245	0.84 ± 0.44	0.75	0.54	0.75	1.06	0	0.17–2.85
80–94	75	0.94 ± 0.52	0.78	0.60	0.78	1.18	0	0.26–2.96
All	1427	0.87 ± 0.41	0.81	0.59	0.81	1.09	0	0.12–2.96
**Men and Women (age)**								
50–60	978	0.97 ± 0.43	0.90	0.65	0.90	1.21	0	0.12–2.93
60–70	895	0.92 ± 0.43	0.86	0.60	0.86	1.16	0	0.11–2.81
70–80	463	0.91 ± 0.45	0.83	0.60	0.83	1.15	0	0.17–2.85
80–94	127	1.01 ± 0.56	0.87	0.61	0.87	1.26	0	0.26–2.96
All	2463	0.94 ± 0.44	0.87	0.63	0.87	1.19	0	0.11–2.96

*SD* Standard deviation

Univariate logistic regression analysis on demographic factors (age, gender, IOP, SE) influencing peripheral ACD showed that gender (*r* = − 0.18, *P* < 0.0001) and SE (*r* = − 0.31, *P* < 0.0001) were significantly associated with peripheral ACD (Table 2). Multivariate logistic regression analysis of the association between peripheral ACD and other ocular parameters (r2 = 0.1304, *P* < 0.0001) revealed that peripheral ACD declined by 0.31 CT (*P* < 0.0001) per diopter of SE and was 0.19 CT (*P* < 0.0001) shallower in women than in men (Table 3).

**Table 2:** Multivariate analysis of the association between peripheral ACD and other parameters

Variable	Nonstandardized Regression Coefficient(95% CI)	Standardized Regression Coefficient	Variance Inflation Factor	*P* value
Intercept	1.34 (1.17,1.51)	0.00	0.00	<.0001
Age	−0.00 (−0.00,0.00)	−0.02	1.02	0.2112
Gender	−0.17 (− 0.20,-0.13)	−0.19	1.01	<.0001
IOP	−0.00 (− 0.01,0.00)	−0.04	1.02	0.0605
SE	−0.04 (− 0.05,-0.04)	−0.31	1.01	<.0001

**Table 3:** Peripheral ACD values and SE in 291 subjects with peripheral ACD less than 0.5 CT in right eyes

Gender	Age	Peripheral ACD			SE		
		n	Mean ± SD	Range	n	Mean ± SD	Range
**Men**	50–60	25	0.36 ± 0.09	0.18–0.49	25	0.93 ± 1.27	−1.00-4.625
	60–70	28	0.37 ± 0.08	0.11–0.47	28	1.00 ± 1.61	−5.375-3.75
	70–80	19	0.39 ± 0.08	0.21–0.48	16	0.09 ± 5.03	−17.375-4.25
	80–94	5	0.43 ± 0.09	0.28–0.49	4	1.22 ± 2.12	−1.625-3.50
	subtotal	77	0.37 ± 0.08	0.11–0.49	73	0.79 ± 2.67	−17.375-4.625
**Women**	50–60	61	0.37 ± 0.10	0.12–0.49	60	0.78 ± 1.03	−3.00-3.75
	60–70	91	0.35 ± 0.09	0.13–0.49	90	0.77 ± 1.53	−8.25-3.875
	70–80	53	0.35 ± 0.09	0.17–0.49	49	0.96 ± 1.65	−4.625-4.125
	80–94	9	0.33 ± 0.07	0.26–0.46	6	2.08 ± 1.43	0.50–4.25
	subtotal	214	0.35 ± 0.09	0.12–0.49	205	0.85 ± 1.44	−8.25-4.25
**All**	50–60	86	0.37 ± 0.09	0.12–0.49	85	0.82 ± 1.10	−3.00-4.625
	60–70	119	0.35 ± 0.09	0.11–0.49	118	0.82 ± 1.55	−8.25-3.875
	70–80	72	0.36 ± 0.09	0.17–0.49	65	0.74 ± 2.84	−17.375-4.25
	80–94	14	0.37 ± 0.09	0.26–0.49	10	1.74 ± 1.68	−1.625-4.25
	total	291	0.36 ± 0.09	0.11–0.49	278	0.84 ± 1.84	−17.375-4.625

Totally, 291 subjects had peripheral ACDs less than 0.5 CT in right eyes. The mean peripheral ACD values for men and women with peripheral ACD less than 0.5 CT in right eyes were 0.37 ± 0.08 (range 0.11–0.49) CT and 0.35 ± 0.09 (range 0.12–0.49) CT respectively (*t* = − 1.46; *P* = 0.1464; Table 4). The mean peripheral ACD in different age groups was not significantly different.

**Table 4:** AUC of different ROC curve and cut-off value

ROC curve	AUC (95% CI)	Cut-off Value	Se	Sp	PV+	Youden Index
peripheral ACD + SE	0.786 (0.726, 0.847)	peripheral ACD =0.360 SE =2.120	0.809	0.670	0.527	0.479
peripheral ACD	0.777 (0.715, 0.839)	peripheral ACD =0.305	0.618	0.862	0.498	0.480
SE	0.547 (0.476, 0.619)	SE =1.188	0.652	0.468	0.346	0.120

*AUC* Area under curve, *Se* Sensitivity, *Sp* Specificity, *PV+* Positive predictive value

A total of 291 subjects with peripheral ACD less than 0.5 CT were made an appointment for gonioscopy in the tertiary eye hospital and 91 PACD subjects were identified, including 6 PACG, 9 PAC and 76 PACS.

**Fig. 2:**
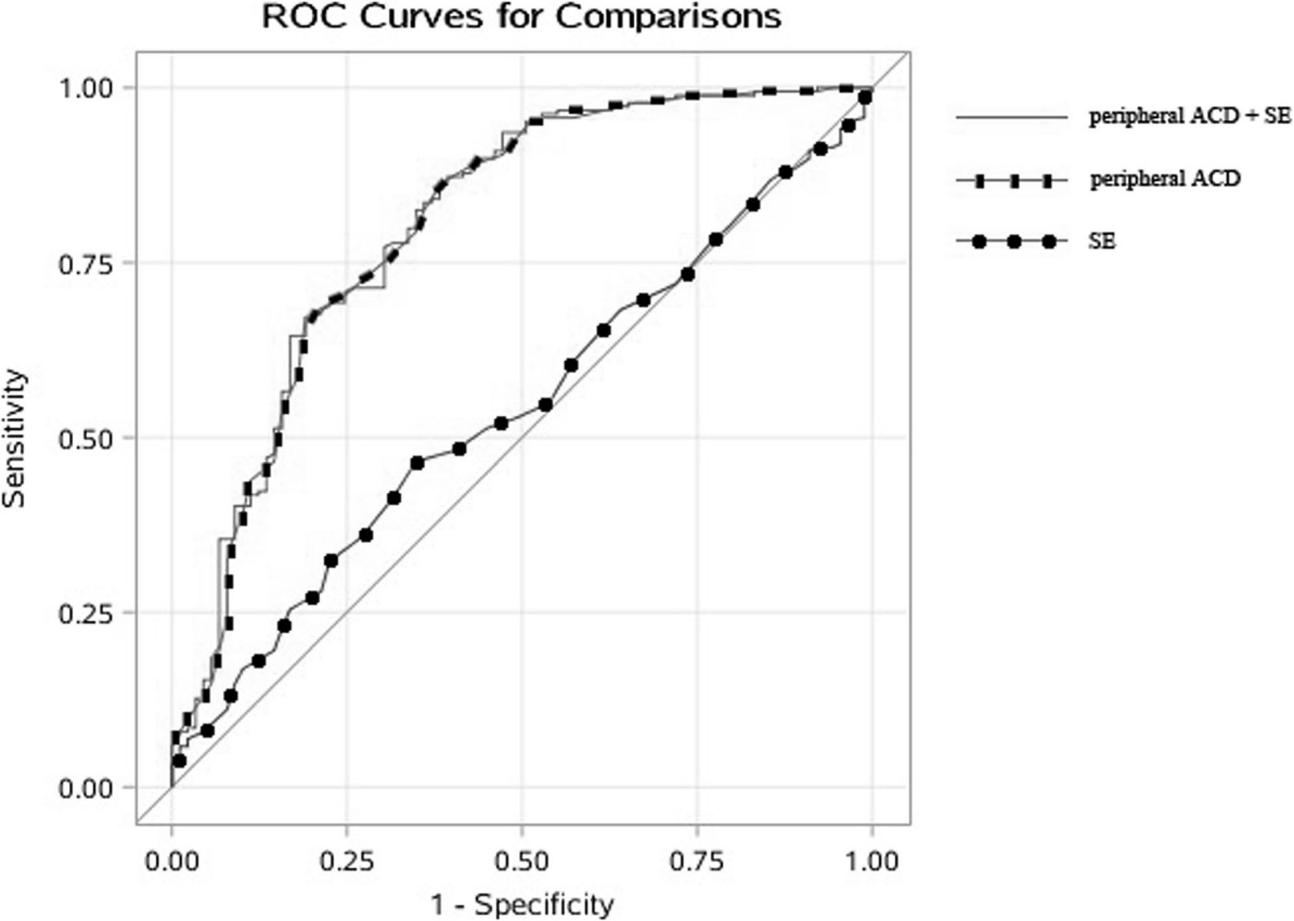
ROC curves of different index for identifying PACD

As gonioscopy was the standard examination for identifying PACD, we compared the efficacy of three screening indexes (peripheral ACD, SE, peripheral ACD combined with SE) with gonioscopy in 291 subjects for identifying PACD (Fig. 2). Using peripheral ACD as screening index achieved sensitivity of 0.618, specificity of 0.862, positive predictive value of 0.498 and Youden index of 0.48 with a cut-off value of 0.305 CT. Using SE as screening index was not significant and the combination of peripheral ACD and SE did not improve screening efficiency (Table 4).

When a modified Van Herrick grading scheme was used to grade the peripheral ACD, the sensitivity, specificity, positive predictive value and Youden index were presented in Table 5. The Youden index was greatest with the cut point of the peripheral ACD grade as 0.3 (Table 5).

**Table 5:** The efficacy of identifying PACD with different peripheral ACD

Peripheral ACD grade	Se	Sp	PV+	Youden Index
< 0.2	0.154	0.995	0.933	0.100
< 0.3	0.571	0.890	0.703	0.403
< 0.4	0.846	0.475	0.423	0.291
< 0.5	1.000	0.000	0.313	0.000

*Se* Sensitivity, *Sp* Specificity, *PV+* Positive predictive value

## Discussion

In this population-based study of Chinese residents of Shanghai, 291 subjects were observed to have a peripheral ACD less than 0.5 CT in their right eyes. Among these subjects, 91 (31.27%, 91/291) subjects were diagnosed as having PACD. In this study, digital remote screening and clinical re-examination for a diagnosis were used at a tertiary eye hospital. Information on all subjects was first collected and analyzed remotely, including visual acuity examination, refraction, IOP measurement, digital anterior slit lamp eye structure photography and digital fundus photography. After this information transmitted to the Shanghai Eye Disease Prevention and Treatment Center, glaucoma suspects were asked permission to proceed with additional and confirmatory reexamination [20].

In this study, the peripheral ACD in 2463 subjects were 0.94 ± 0.44 CT, and 11.8% subjects had reported a peripheral ACD < 0.5 CT. The population distribution of peripheral ACD has been studied in Europe, North America, and Asia [22–24].

Based on peripheral anterior chamber depth, Van Herrick classification has been used to detect angle closure glaucoma (ACG) eyes [25]. This method has proved to be effective and suitable for glaucoma screening. However, Van Herrick’s classification only had five categories (0, 1/4, 1/2, 3/4, 1 CT). The Van Herrick’s classification has been suggested to have low sensitivity and specificity to detect patients with ACG [11]. Studies in southern India, and Greenland reported suboptimal performance of this test in screening for PAC [26, 27]. Therefore, in this study, a modified Van Herrick grading scheme was performed with eight categories (0, 0.05, 0.15, 0.25, 0.35, 0.45, 0.75, and ≥ 1.0 CT) instead of the usual five categories. These values were chosen to give class limits of 0, < 0.1, < 0.2, < 0.3, < 0.4, < 0.5, < 1.0, and ≥ 1.0 CT. This technique has been demonstrated to be valid in other studies [28–30].

Our research shows that the peripheral ACD declines by 0.31 CT per diopter of SE and was 0.19 CT shallower in women than in men. Our findings agree with previous studies that women are more likely to have a shallower peripheral ACD [31]. Similar to other reports, our results are consistent with hyperopic patients likely to have shallower ACDs [32].

The present study used three different indexes (SE, peripheral ACD, SE combined with peripheral ACD) to investigate the most efficient way for PACD screening. The independent SE as the screening index for PACD was not statistically observed and did not significantly improve the screen capability of peripheral ACD for PACD, too. On the basis of these findings, only peripheral ACD measurement should be used to PACD screening in community elderly Chinese.

In our research, when peripheral ACD was < 0.3 CT, Youden index performed best in all groups and the positive predictive value was 0.703, however, a higher sensitivity is needed for effective community screening. The use of a CT cut off value of 0.4 would achieve much higher sensitivity (0.846). Our findings disagree with those from North America, Greenland, and Australia, which reported a cut-off value of 0.25 CT [26, 28, 33]. The reasons may be that the pathogenisis of angle closure is different in different population and the assessment method of peripheral ACD in this study is different from that in other studies. Similarly, Foster et al. also reported the augmented Van Herrick scheme offers enhanced performance in detection of established PACG [29].

A limitation of this study is that only subjects with peripheral ACD < 0.5 CT were asked to undergo gonioscopy, which may give rise to a potential cause of bias. Three major rationales were used for our screening method design. First, according to Van Herrick, a subject with a peripheral ACD ≥0.5 CT would have a very low probability of having angle closure [25]. Second, there was a study in Australia, in which goniscopy was carried out only with peripheral ACD ≤ 0.3 CT [28]. Third, Foster et al. performed goniscopy on subjects and indicated that in subjects witth peripheral ACD ≥0.5 CT, no patients had closed angles. Moreover, when the cut off of < 0.3 CT with peripheral ACD was used in this study, the Youden index was still not particularly high, which called a measure with a higher sensitivity for community screening of glaucoma. Therefore, a peripheral ACD < 0.5 CT is likely to be a relatively cost-effective index for glaucoma screening, although we do not recommend that only the estimation of peripheral ACD be used instead of gonioscopic examination in patients suspected of glaucoma.

## Conclusions

Peripheral ACD measurement is recommended for PACD screening in community elderly Chinese. Further research is still required for finding out the most efficient screening technique for primary angle closure disease in China.

## Data Availability

The datasets used and analysed during the current study are available from the corresponding author on reasonable request.

## Abbreviations

ACD: Anterior chamber depth
SE: Spherical equivalent
IOP: Intraocular pressure
CT: Corneal thickness
ROC: Receiver operating characteristic curves
PACD: Primary angle closure disease
PACG: Primary angle closure glaucoma
POAG: Primary open angle glaucoma
RNFL: Retinal nerve fiber layer
OCT: Optical coherence tomography
VCDR: Vertical cup to disk ratio
PAS: Peripheral anterior synechiae
